# Poor housing quality and the health of newborns and young children

**DOI:** 10.1038/s41598-024-63789-z

**Published:** 2024-06-05

**Authors:** Tamás Hajdu, Gábor Kertesi, Bence Szabó

**Affiliations:** 1 Institute of Economics, HUN-REN Centre for Economic and Regional Studies, Budapest, Hungary; 2 Corvinus University of Budapest, Budapest, Hungary

**Keywords:** Risk factors, Health policy, Public health, Epidemiology

## Abstract

This study uses linked administrative data on live births, hospital stays, and census records for children born in Hungary between 2006 and 2011 to examine the relationship between poor housing quality and the health of newborns and children aged 1–2 years. We show that poor housing quality, defined as lack of access to basic sanitation and exposure to polluting heating, is not a negligible problem even in a high-income EU country like Hungary. This is particularly the case for disadvantaged children, 20–25% of whom live in extremely poor-quality homes. Next, we provide evidence that poor housing quality is strongly associated with lower health at birth and a higher number of days spent in inpatient care at the age of 1–2 years. These results indicate that lack of access to basic sanitation, hygiene, and non-polluting heating and their health impacts cannot be considered as the exclusive problem for low- and middle-income countries. In high-income countries, there is also a need for public policy programs that identify those affected by poor housing quality and offer them potential solutions to reduce the adverse effects on their health.

## Introduction

Given that health at birth and in early childhood plays an important role in shaping later life outcomes^1–11^, a large body of research has studied the different factors influencing various indicators of newborns’ health and has examined the extent of inequalities. Numerous studies have shown that there are huge disparities in the health status of newborns and young children around the world^12–24^. It is a common worldwide phenomenon that disadvantaged newborns tend to have a lower birth weight and are more likely to be born prematurely. They also have worse early childhood health indicators than their more advantaged peers. A concerning fact is that many of these differences appear to be stable over time rather than decreasing in any significant way^18,19,21,22^.

Reducing the often immense health inequalities in infant and early childhood is important not only for moral and social justice reasons but also for economic reasons. Interventions at younger ages can generate high cumulative benefits by altering developmental trajectories, and they are usually more cost-effective compared to interventions at older ages, especially if they are effectively targeted^25–27^. Policies that can improve the health of the most vulnerable children not only benefit them but are also likely to generate significant social benefits by reducing (current and future) negative externalities from inequalities.

Lack of access to good quality water, sanitation, and hygiene (WASH) and exposure to polluted air can be an important reason for poor children's lower health status. A large body of literature focuses on these issues and analyzes the impact of low housing quality, access to basic sanitation, poor hygiene, and indoor air pollution (from polluting fuels used for cooking and heating), which are serious problems for hundreds of millions of children. Several studies have concluded that these factors are important contributors to poor health—both at birth and in early childhood—including low birthweight, preterm birth, small for gestational age, respiratory infections, pulmonary diseases, infectious diseases, and infant and child mortality^28–43^. Although mortality associated with household air pollution has substantially decreased in the last decades, a recent paper estimated that even in 2018, 320,000 deaths of children could be attributed to household air pollution from the use of solid fuels in low-income and middle-income countries^44^. Another study calculated that in children under five years of age, almost 300,000 diarrheal deaths were attributable to inadequate WASH in 2016^45^. This figure has only declined slightly by 2019^46^. In addition, 112,000 deaths from acute respiratory infections were attributed to unsafe hygiene in this age group in 2019^46^. Without significant future policy interventions, these figures are unlikely to change much. The prevalence of solid fuel use is expected to remain sizable in the future: a recent study projects that almost one in three people will still be using polluting solid fuels in 2030^47^. Besides, access to safe drinking water and adequate sanitation facilities will continue to be a challenge for many people around the world in the near future^48^.

These issues are often seen as the exclusive problem of low- and middle-income countries, and although their prevalence is significantly higher in these countries, poor and disadvantaged people in high-income countries also suffer from the consequences of poor sanitation and indoor air pollution^49–52^. Low-income households and minorities, like the Roma in Europe, especially lack access to adequate WASH^53,54^, and Hungary is no exception^55^.

In this paper, we advance the literature on the effect of inadequate WASH and indoor air pollution using data from Hungary. Hungary is an interesting case: despite universal access to healthcare, health inequalities persist and have even increased over the past 30 years, and are one of the largest in Europe^19,56,57^. Homeownership rate is very high, and at the same time there is a significant shortage of social rental housing^58–60^. In a result, residential mobility is very low in a European perspective^60,61^. Although a major rehabilitation program of urban housing estates built with prefabricated technology started in the 2000s, large-scale housing renovation programs targeting the worst-quality (mostly rural) homes have not been implemented in the last few decades^58,59^.

We construct a large and unique, individual-level dataset of children born in Hungary between 2006 and 2011 by linking three administrative datasets: birth records, population census, and administrative data of inpatient care. We examine six indicators of health at birth and three indicators of inpatient care in early childhood and analyze their relationship with an index of poor housing quality. This housing quality index includes the lack of access to piped water, bathrooms, hot running water or toilets, exposure to polluting heating, and adobe houses. In our analysis, we control for many aspects of parental background, including education, age, ethnicity, labor market status, occupation, and marital status, along with the mother's pregnancy history (live births, induced abortions, and fetal losses). Importantly, we also control for the unobserved characteristics of small geographic areas that uniformly influence the health of the newborns and young children living there by introducing census tract fixed effects.

Although it is beyond the scope of this study to identify the potential mechanisms, the literature suggest that one of the main biological pathways through which poor hygienic and sanitation conditions can affect intrauterine development and early childhood health is the higher prevalence of genitourinary infections^62,63^. Poor hygienic and sanitation conditions are also associated with early childhood lower respiratory tract infections (e.g. RSV) that even predispose children to premature adult death from respiratory diseases^64^. Maternal air pollution has been linked to preeclampsia, gestational hypertension and placental growth^65,66^, which can lead to complications in pregnancy, low birth weight and premature birth. The detrimental effects of air pollution for fetal development are attributable to a large extent to the use of solid fuels in cooking and heating^67^. Finally, adobe houses of poor people in Hungary are frequently built without foundation and insulation^68^. As a result, damp patches and mold appearing on the walls trigger early childhood allergic responses, influence respiratory health, and increase hospital admissions^69–71^.

We find in this paper that exposure to poor housing quality in the fetal period is associated with significantly lower birth weight, shorter gestation, lower APGAR score, and a higher chance of being an SGA (small for gestational age) infant. In addition, extremely poor housing quality is also correlated with a higher number of days spent in inpatient care at the age of 1–2 years, even when controlling for indicators of health at birth.

Our analysis contributes to the existing literature in at least three ways. First, unlike the previous literature, we use linked administrative data. One of the advantages of this dataset is that the number of observations is exceptionally large compared to usual survey data (more than 250,000), therefore the statistical power of our study is high, and relatively low-prevalence phenomena can be studied (such as the health impacts of extremely low-quality housing in high-income countries). Administrative data are also usually more accurate than survey data as they result from standard measurements and data collection, and they are not affected by the accuracy of individuals' recalls and misreporting. On the other hand, the shortcoming of administrative data is that they are not collected for research purposes, so they often do not contain some important information that may be relevant for research. Second, we provide evidence on the relationship between housing quality and the health of newborns and young children in a high-income country. Most of our knowledge is from developing countries, but as we noted earlier, poor housing quality is also experienced by many disadvantaged people in high-income countries. Because of the much more universal access to quality health care, prenatal screening and care programs, it is not obvious that the relationship between poor housing quality and newborn health should be the same in high-income countries as it is in low-income countries. Third, we examine both health at birth and in early childhood, so we can gain some insight into which periods are more affected by poor housing quality. Previous studies could not make such comparisons because they focused on the health of either newborns or young children.

The rest of the paper proceeds as follows. "Data and methods" describes the data and methods used in the paper. "Results" presents the results. "Discussion" discusses the implications of the findings and concludes.

## Data and methods

### Live birth records

The first administrative dataset of this analysis is the live birth registry of the Hungarian Central Statistical Office (HCSO). This de-identified dataset covers all live births in Hungary from 1970 onwards, and, among others, it contains information on several birth-related variables: the date of birth, gestational age (determined by the first day of the last menstrual period), birth weight, birth length, and APGAR score at 5 min after birth (APGAR score evaluates the health condition of the newborns using five criteria—Appearance, Pulse, Grimace, Activity, Respiration—and ranges from 0 to 10). It also includes information on the mother’s and father’s age, education, labor market status, occupation (Hungarian standard classification of occupations), and the municipality of residence. The marital status and the pregnancy history of the mother are also known (number of previous live births, induced abortions, and spontaneous pregnancy losses).

We defined six outcome variables that capture a newborn’s health at birth: (i) birth weight (measured in grams), (ii) an indicator of low birth weight (birth weight < 2500 g), (iii) gestation length (measured in completed weeks), (iv) an indicator of preterm birth (pregnancy length < 37 weeks), (v) low APGAR score (≤ 8), and (vi) an indicator of small for gestational age (SGA).

### Population census

Information on housing quality comes from the 2011 population census of the HCSO. The anonymized dataset available for research purposes covers the entire population of Hungary. The 2011 census was conducted in October 2011, the reference date was October 1st, 2011. In addition to the information on individuals, the census also included a separate housing questionnaire, which measured both the characteristics of the dwelling and how long the respondent had lived there. We defined six binary indicators of poor housing quality: (i) lack of flush toilet, (ii) lack of a bathroom, (iii) lack of piped water, (iv) lack of hot running water, (v) adobe house, (vi) polluting heating. Heating with solid fuels (wood or coal) was considered to be a polluting heating method if each room was heated separately. These six variables were summed to create an index of poor housing quality, ranging from 0 to 6. A value of 0 means that the dwelling is not considered to be of poor quality in any of the aspects assessed, whereas a value of 6 indicates the worst quality dwellings, i.e., the dwelling is considered to be of poor quality according to all the indicators assessed.

Beyond housing quality, the Roma ethnicity of the parents is also derived from the 2011 census. The Roma are one of the largest and poorest ethnic minorities in Europe. In Hungary, it is estimated that more than 8 percent of the total population is Roma^72^. They face poverty, multiple disadvantages, and discrimination^73–79^. Ethnicity was measured by two questions, allowing for multiple identities. All mothers and fathers were categorized as Roma if they identified themselves as Roma in either of the questions on ethnicity. Information from the 2011 census also allows us to take into account the characteristics of the geographical micro-environment of the children. The smallest unit of the neighborhood in the Hungarian census is the census tract containing around 250 individuals on average. Each census respondent belongs to a census tract.

### Inpatient care

The health care system in Hungary is single-payer system. The vast majority of individuals are insured, inpatient and outpatient care is financed by compulsory health insurance and is free of charge. Total opt-out is prohibited, but people can use private care for certain services. This is typically the case for outpatient services, but even there it is a small number of cases. Inpatient care in private care is most common for obstetric care. Private inpatient care for young children is practically non-existent.

Anonymized data on inpatient care are obtained from the medical records of the Hungarian National Healthcare Services Center (NHSC). For the period 2008–2017, we have information on all inpatient stays for children born between 2008 and 2016 in public healthcare. Inpatient care events can be transformed into a panel database using an anonymized identifier. For each event, the patient's sex, date of birth, place of residence (zip code), and the date of the care event are known. Specific health conditions can be identified by the International Classification of Diseases (ICD) codes. We focus on inpatient care at the age of 1–2 years, as the anonymized identifier may have changed during the first few weeks/months of life due to administrative reasons.

From the inpatient care records, we created three indicators of early childhood health: (i) the number of days spent in inpatient care for any disease (ICD codes: A00-Z99), (ii) the number of days spent in inpatient care for respiratory diseases (ICD codes: J00-J99), and (iii) the number of days spent in inpatient care for infectious diseases (ICD codes: A00-B99). Each of these shows the total number of days spent in hospitals over the two years from age 1 to the end of age 2.

### Data linkage and sample selection

The population used to study the relationship between housing quality and health at birth consists of singleton births in the live birth dataset between September 2006 and August 2011. In the first step, we excluded births with missing information on health at birth. Next, the birth records were linked to the census data. Neither birth records nor census records contain any personal identifiers, such as social security numbers, that would help link them. The main variables used for the linkage are the exact date of birth of the child and mother, the sex of the child, and the place of residence of the mother at the time of the child's birth. We found some additional matches when we narrowed down the multiple matches by including other variables (father’s birth date, and parents’ education). In the linked dataset, we excluded records where moving into the census dwelling occurred after the start of pregnancy. (This was possible because one of the questions in the census asks how long the respondent has lived in the current dwelling.) Finally, records where any item of the housing quality index was missing were excluded. The number of observations excluded at each step of the sample selection is reported in Table A1 (Online Appendix A). While problems of missing key variables only occur in the case of 1–3% of observations, we lose around 10% of the sample at linking the birth registry to the census and around 30% when we exclude those who have moved since the beginning of their pregnancy. This, however, is a necessary step as we want to ensure that the housing conditions derived from the census characterize the mother’s living conditions while pregnant. The final sample covers 253,929 children.

When we analyze associations with early childhood health, we have to work with a narrower sample that includes children born between January 2008 and August 2011. Beyond the data linkage steps described above, in this case, we require successful linkage to the inpatient care data, which results in excluding around 27% of the relevant original sample (Table A2, Online Appendix A). The main reason for this relatively high failure rate is that we were only able to use the following information to link inpatient care data: date of birth, sex, and place of residence. The final sample, which is used to examine the relationship between housing quality and early childhood health, consists of 107,934 children.

We can form an understanding of the introduced bias by examining the evolution of the key outcome variables over the steps of the sample selection, which we report in Table A3 (Online Appendix A). The magnitudes of the induced differences are small. For instance, the final analysis sample has an around 20 g higher mean birth weight than the starting singleton dataset, so the final analysis sample contains information on children with slightly better health outcomes on average. Additionally, the observations for the analysis of early childhood health are even closer concerning health outcomes to the original sample: in terms of birth weight, the difference is only around 10 g. As we control for several observable characteristics in the regressions and the selection does not seem to impact the key outcomes substantially, it is likely that our results are not far from what we would estimate for the entire population. Nevertheless, we re-estimated our main results with inverse probability weighting in our robustness checks. In this exercise, the weights are derived from a probability model that runs on the baseline dataset (singleton births in the live birth registry) and predicts the probability of being included in the final analysis samples with all information available in the birth records.

Table 1 shows the descriptive statistics of the outcome variables and the index of poor housing quality for the two analysis samples of our study. The average birth weight is somewhat more than 3300 g, while the average gestation length is nearly 39 weeks. Around 6% of the newborns were born with a low birth weight (< 2500 g) or premature (before the 37th week of pregnancy), 5% of the sample have a low APGAR score, and the share of SGA newborns is almost 10%. The children in the early childhood health sample spent, on average, nearly two days in hospital between the ages of 1–2 years. Nearly one hospital day was for respiratory illnesses and 0.6 days for infectious diseases. The average score of the poor housing quality index is 0.5 in the health at birth sample and slightly higher (0.6) in the early childhood health sample. The descriptive statistics of the control variables are shown in Table A4 (Online Appendix A).

**Table 1:** Descriptive statistics.

Variable	Mean	SD	N
Birth weight	3327	541	253,929
LBW	0.057	0.233	253,929
SGA	0.098	0.297	253,929
Gestation length	38.85	1.71	253,929
PTB	0.065	0.247	253,929
Low APGAR	0.052	0.222	253,929
N of days in inpatient care (any diseases)	1.90	7.39	107,934
N of days in inpatient care (respiratory diseases)	0.90	4.21	107,934
N of days in inpatient care (infectious diseases)	0.59	3.27	107,934
Poor housing quality index (health at birth sample)	0.52	1.20	253,929
Poor housing quality index (early childhood health sample)	0.61	1.27	107,934

### Methods

The association between poor housing quality and the health of newborns and young children is estimated by the following regression:

1 $${H}_{iymc}={\beta PHQI}_{iymc}+\gamma {{\varvec{X}}}_{iymc}+{\rho }_{ym}+{\tau }_{c}+{\varepsilon }_{iymc}$$

where H is the health of child *i*, born in year *y* and month *m*, and living in census tract *c*. PHQI is the index of poor housing quality (ranging from 0 to 6), and β shows how one higher value of the index is associated with lower/higher health. When the outcome variables are health at birth, β indicates the influence of housing quality during pregnancy. When the outcome variables are early childhood health, β indicates the joint influence of housing quality during pregnancy and early childhood.

X denotes the vector of control variables. It includes the sex of the child, the mother’s and father’s age (13–17, 18–24, 25–29, 30–34, 35–39, 40–), education (primary or less, vocational, high school, tertiary), labor market status (employed, unemployed, on maternity leave, student, other), Roma ethnicity, and occupation (Hungarian standard classification of occupations codes). For the mothers, the marital status (single, married, divorced, widowed), the number of previous live births (0, 1, 2, 3, 4, 5 +), induced abortions (0, 1, 2, 3 +), and spontaneous pregnancy losses (0, 1, 2, 3 +) were also considered. For the occupation codes, the most detailed categories were considered. The classification system distinguishes nearly 500 occupations. For each of these four-digit occupation codes, a binary indicator variable was included in the regressions, allowing us to control for the effect of occupation on health in the most flexible way. Missing dummies for all control variables are also included.

Year-by-month fixed effects (ρ) control for those unobserved factors that uniformly affect the health of children born in the same year and month. Census tract fixed effects (τ) control for all unobserved location-specific factors that do not change over the years studied and affect the health of children living in the same small neighborhood (e.g., quality and availability of outpatient and GP care in the neighborhood, quality of drinking water, etc.).

## Results

### Prevalence of low housing quality

**Figure 1:**
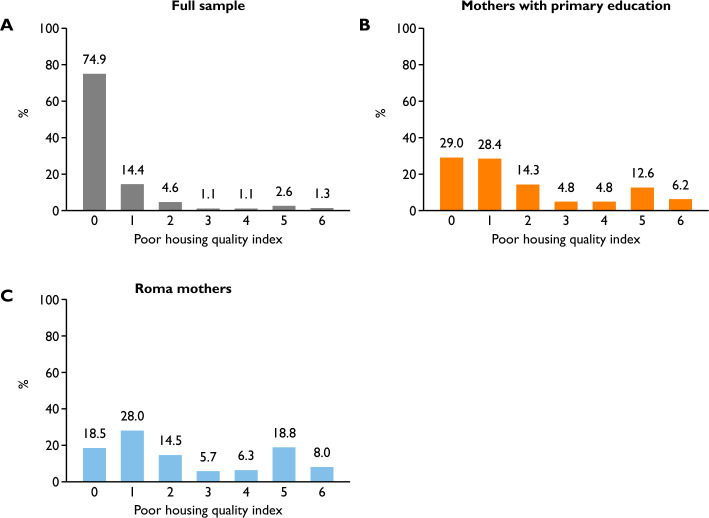
Distribution of observations by values of the index of poor housing quality. In the health at birth sample. (**A**) N = 253,929, (**B**) N = 44,388, (**C**) N = 17,552.

Poor-quality housing is not an uncommon phenomenon among children in Hungary (Fig. 1). One-quarter of the children in the health at birth sample live in a home that does not meet at least one of the basic quality criteria we examined, and 4% of children live in a home that scores 5 or 6 on the poor housing quality index. The latter children lack access to basic sanitation facilities such as piped water, flush toilets, or bathrooms, and their homes are characterized by a heating system that is considered polluting. These results are qualitatively similar when examining the distributions in the early child health sample (Fig. A1, Online Appendix A).

Importantly, these rates are significantly worse for disadvantaged children. 19% of children of mothers with at most primary education live in very poor-quality housing (index scores 5–6), while only 29% live in a home that is not considered poor quality on any of the indicators assessed. For children of Roma mothers, these figures are 27% and 19%, respectively. These deprived groups represent significant segments of society. Children of mothers with at most primary education account for 16% of the sample, while children of Roma mothers account for 6%. These results clearly show that poor-quality housing can be quite widespread among the poorest members of society, even in a developed country like Hungary.

Figure A2 in the Online Appendix shows the prevalence of the components of the poor housing quality index. Polluting heating and homes made of adobe are the two most common quality problems, affecting 17.5% and 13.1% of children respectively. However, the prevalence of the other components is also not negligible, ranging from 3.1 to 6.7%.

**Figure 2:**
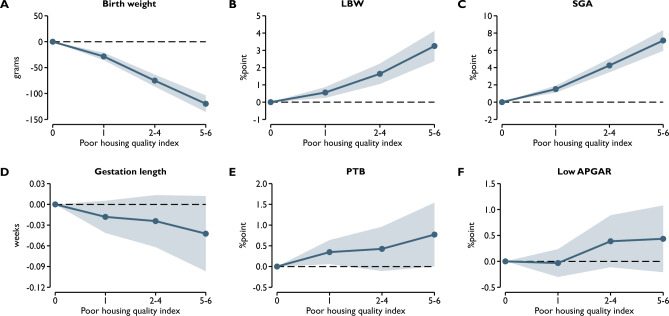
Housing quality and health at birth. The reference category is 0. Shaded areas represent 95% confidence intervals. Control variables, year-by-month fixed effects and census tract fixed effects are included.

### Health at birth

Table 2 summarizes the estimated associations between poor housing quality and health at birth estimated using Eq. (1). The results show overall that the poorer the housing quality, the worse the health of newborns. A one-point higher index value is associated with a 24-g lower birth weight and a 0.64 percentage-point increase in the chance of being born with a low birth weight. In terms of gestation length, a one-point higher index value of poor housing quality is associated with a 0.01-week shorter pregnancy length and a 0.18 percentage point higher chance of preterm birth. A one-point higher index value is also associated with a 1.4 and 0.1 percentage point higher chance of being born as a newborn with SGA and a low APGAR value, respectively.

**Table 2:** Housing quality and health at birth.

	(1)	(2)	(3)	(4)	(5)	(6)
	Birth weight	LBW	SGA	Gestation length	PTB	Low APGAR
Poor housing quality index	− 24.39*** (1.37)	0.0064*** (0.0007)	0.0140*** (0.0010)	− 0.0106** (0.0046)	0.0018** (0.0007)	0.0011* (0.0006)
N of obs	253,929	253,929	253,929	253,929	253,929	253,929
R-squared	0.128	0.070	0.082	0.071	0.057	0.275
Controls	Yes	Yes	Yes	Yes	Yes	Yes
Census tract FE	Yes	Yes	Yes	Yes	Yes	Yes
Year-by-month FE	Yes	Yes	Yes	Yes	Yes	Yes

Controls: sex of the child, the highest level of education, labor market status, occupation code, ethnicity, and age of the mother and father, marital status of the mother, number of previous live births, induced abortions, and spontaneous fetal losses of the mother. Robust standard errors are in parentheses. ***p < 0.01, **p < 0.05, *p < 0.1.

These values are especially substantial when comparing children with minimum (0) and maximum (6) index values. The difference is 146.3 g for birth weight, 3.8 percentage points for LBW, 8.4 percentage points for SGA, 0.06 weeks for gestation length, 1.1 percentage points for PTB, and 0.7 percentage points for low APGAR.

The sensitivity of the results is explored by a series of robustness tests: different location fixed effects, the inclusion of additional control variables, weighting, and the use of a narrower sample. First, we experimented with ZIP code fixed effects instead of census tract fixed effects (Table A5, Online Appendix A). Second, we added further control variables that describe the household composition and characteristics in the 2011 census (Table A6, Online Appendix A). These were the number of household members of different ages, the proportion of employed and unemployed persons among 25–59-year-olds living in the household, the proportion of tertiary and secondary education among 25–59-year-olds, the proportion of people speaking foreign languages (English, German) among 25–59-year-olds, the proportion of people with long-lasting disease or impairment among 25–59-year-olds, and floor space per inhabitant in the dwelling. These additional control variables help us to capture more accurately those permanent socioeconomic circumstances of the children’s household that may have shaped their health at birth and may be correlated with poor housing quality. Third, we re-estimated the regressions with weights that represent the inverse probability of being included in the final analysis samples (Table A7, Online Appendix A). Finally, we restricted the sample to children born between September 2008 and August 2011 (Table A8, Online Appendix A). In this way, we were trying to ensure that housing quality measured in 2011 describes as accurately as possible the housing conditions in the fetal period. Due to housing renovations, the housing conditions in the fetal period may, in some cases, differ from the 2011 situation, but by narrowing the time window this risk is reduced. The main results are robust, none of these changes alter the conclusions. Poor housing quality is associated with lower health at birth in all specifications.

Similar results are obtained when, instead of simply summing the six indicators of poor housing, we calculate our index by summing the z-scores of the six items (Table A9, Online Appendix A). The strength of the associations is similar to the baseline result when interpreting the coefficients for a change of one standard deviation. For example, a one standard deviation increase in the poor housing quality index is associated with a 29.3 g lower birth weight in the baseline approach (24.39 × 1.2), and a 27.4 g lower birth weight when the index is calculated using the z-scores (5.96 × 4.6). In addition, significant associations are observed even when the continuous outcome variables are log-transformed (Table A10, Online Appendix A).

Next, we examined the potential nonlinearity of the relationship between poor housing quality and the indicators of health at birth. The seven values of the index of poor housing quality are grouped into four categories to ensure that the categories have a sufficient number of observations and that the standard errors are not too large. The categories are 0, 1, 2–4, and 5–6 index values. Figure 2 summarizes these results. We can see that the estimated relationships can be considered mostly linear. Even at low index values, the health indicators of newborns are worse, and the marginal effects appear to be roughly constant. For some outcome variables, however, the estimates are quite noisy, so the coefficients cannot be considered statistically significant. Nevertheless, the general trend still holds in these cases as well.

Finally, we estimated regressions using the six indicators of housing quality instead of the index of them (Fig. A3, Online Appendix A). These results show, not surprisingly, that the total value of the six coefficients is about six times the estimated coefficient of the housing quality index obtained by summing them (see Table 2). Although the standard errors of the individual coefficients are sometimes large, the point estimates suggest that lack of access to hot water, flush toilet, and bathroom, and polluting heating are the most strongly associated with most indicators of health at birth. For example, in the case of birth weight, lack of access to hot water is associated with a 48.6-g lower birth weight, while for polluting heating, lack of access to bathroom, and lack of access to flush toilet, the estimated coefficients are − 47.5, − 21.1, and − 16.9, respectively. However, for other outcome variables, the relative strength of associations may differ.

**Figure 3:**
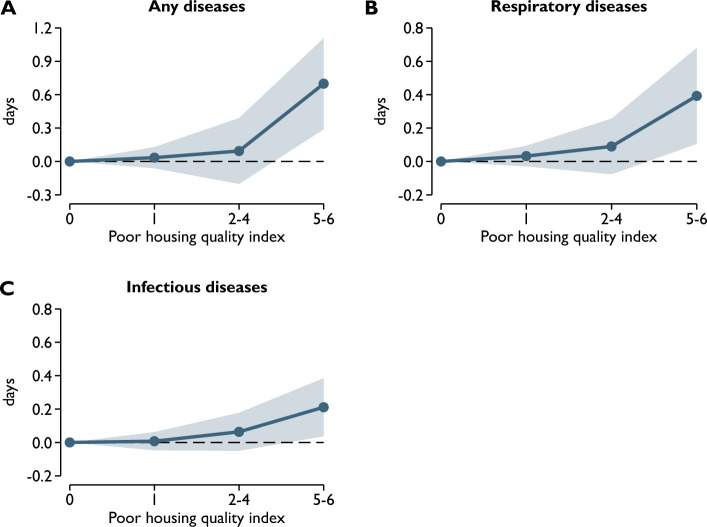
Housing quality and early childhood health. The dependent variables are the number of days spent in inpatient care at age of 1–2 years. Respiratory diseases = ICD-10 codes J00-J99. Infectious diseases = ICD-10 codes A00-B99. The reference category is 0. Shaded areas represent 95% confidence intervals. Control variables, year-by-month fixed effects and census tract fixed effects are included.

### Early childhood health

The relationship between poor housing quality and early childhood health is summarized in Table 3. The results show that children living in poor-quality homes spent more time in hospitals than children living in good-quality homes. A one-point higher value of the index of poor housing quality is associated with 0.11 more days spent in inpatient care at age 1–2 years. The results in Column 2 and Column 3 of Table 3 suggest that this overall increase is mainly due to an increase in hospital stays for respiratory and infectious diseases. A one-point higher index value is associated with 0.07 and 0.03 more hospital days for respiratory and infectious diseases, respectively.

**Table 3:** Housing quality and early childhood health.

	(1)	(2)	(3)
	Any diseases	Respiratory diseases	Infectious diseases
Poor housing quality index	0.108*** (0.036)	0.067** (0.026)	0.034** (0.015)
N of obs	107,934	107,934	107,934
R-squared	0.081	0.092	0.077
Controls	Yes	Yes	Yes
Census tract FE	Yes	Yes	Yes
Year-by-month FE	Yes	Yes	Yes

The dependent variables are the number of days spent in inpatient care at the age of 1–2 years. Respiratory diseases = ICD-10 codes J00-J99. Infectious diseases = ICD-10 codes A00-B99. Controls: sex of the child, the highest level of education, labor market status, occupation code, ethnicity, and age of the mother and father, marital status of the mother, number of previous live births, induced abortions, and spontaneous fetal losses of the mother. Robust standard errors are in parentheses. ***p < 0.01, **p < 0.05, *p < 0.1.

As earlier, the robustness of the results is tested by using alternative location fixed effects (Table A11, Online Appendix A), including further control variables (Table A12, Online Appendix A), and applying weights that correct for the non-random chances of being selected in the sample (Table A13, Online Appendix A). The main conclusions remain unchanged in all these specifications. We also estimated regression using the sum of the z-scores of the six housing quality items. If the coefficients are interpreted for a change of one standard deviation, the results of a regression using the z-scores are virtually identical to the results of the baseline approach (Table A14, Online Appendix A).

In addition, a new specification is estimated in which indicators of health at birth are controlled for (Table A15, Online Appendix A). Since the estimated coefficients in this specification are only slightly lower than in the baseline regressions, it can be concluded that the association between poor housing quality and early childhood health is not simply due to worse health at birth but that the exposure to poor hygiene and sanitation in the early years plays an independent role.

Examining the potential nonlinearity of the relationship between poor housing quality and early childhood health, we find that the relationship is rather nonlinear (Fig. 3). Large differences are observed between children living in very poor-quality homes (index score 5–6) and children living in good-quality homes (index score 0). The difference in hospital days for any disease is 0.7 days, whereas for respiratory and infectious diseases, it is 0.4 and 0.2 days, respectively. However, the early childhood health indicators of children with index scores of 1–4 are not particularly different from those of children with an index score of 0.

When we use the six indicators of housing quality instead of our poor housing quality index, we find that lack of access to piped water is the most strongly associated with the number of days spent in hospitals (Fig. A4, Online Appendix A). But it should be noted that some items are highly correlated, which makes it more challenging to interpret the results correctly, therefore our preferred specification is the one that uses the poor housing quality index.

## Discussion

By linking birth certificates, census records and administrative data of inpatient care for children born in Hungary between 2006 and 2011, this paper addressed the question of how poor housing quality is associated with health at birth and in early childhood. Unlike most of the previous literature, it used data from a high-income country and showed that although the average standard of living is high in Hungary, poor housing quality is not at all a marginal problem, especially among disadvantaged children. It is worth pointing out that the index of poor housing quality includes, among other things, the lack of access to basic sanitation requirements such as a bathroom, running water, or a flush toilet in the home. One might think that these problems were almost non-existent in the 2010s in a member state of the European Union—especially since adequate housing was recognized as a fundamental human right in the Universal Declaration of Human Rights as early as 1948—but we showed that the vast majority of poor people have low-quality housing on at least one criterion. In fact, a fifth of children of mothers with at most primary education and a quarter of children of Roma mothers live in extremely poor-quality homes, characterized by a lack of piped water, flush toilets, bathrooms, adobe walls, and polluting heating. This means that the potential impacts should not be considered as a marginal issue but as a substantial public policy problem.

We showed that poor housing quality is associated with lower health at birth and a higher number of days spent in inpatient care at the age of 1–2 years. Importantly, the estimated health differences are especially immense when comparing children with minimum and maximum index values of housing quality. It is also important to note that in the case of early childhood health, the associations seem to be nonlinear. Only children with extremely low housing quality spend more time in hospitals than children living in good-quality homes. No difference is observed for those with lower index scores.

Direct comparisons of our results with previous literature are difficult because they usually examine different indicators of housing quality, and early childhood health indicators also differ. We have analyzed data from a high-income country, which means that the results may be different in lower-income countries, where poor quality housing is likely to have different specific characteristics. For example, solid fuel heating (and cooking) is likely to represent much higher levels of air pollution in lower-income countries. This may be one reason why exposure to indoor air pollution from solid fuel use is more strongly associated with birth weight in these countries than in Hungary^80^. At the same time, the importance of housing quality is indicated by the fact that our estimated differences in birth weight and LBW rates (between children with low and high index scores) are very similar to the black–white differences^81,82^ (adjusted for socio-economic and behavioral factors). Some papers have also found similar differences between newborns from immigrant and non-immigrant backgrounds in high-income countries^83,84^, but in this case the pattern is less clear and the results are mixed, mainly due to selection^85–87^.

Housing quality seems to be more important for some outcomes than for others. Calculating the health differences, in terms of standard deviation, between children living in the worst-quality homes and children living in good-quality homes reveals that the difference is particularly large for birth weight, LBW, and SGA. For these variables, the estimated differences are between 0.16 and 0.28 standard deviation (Table A16, Online Appendix A). For gestation length, PTB, and low APGAR the differences are around 0.03–0.04 standard deviation, whereas for the indicators of early childhood health, they are 0.06–0.10 standard deviation (Table A17, Online Appendix A).

It is worth pointing out that the relationship between housing quality and children's health was examined while controlling for local neighborhoods. The census tract fixed effects allowed us to control for any unobserved, place-specific factors that uniformly affect the health of all children living in the same small area. This means that our estimates do not include the impact of exposure to poor-quality housing in the neighborhood, which can also have a significant impact on the health of newborns and young children^88–90^.

Although at first glance the recommendations and policy conclusions from the results seem clear, i.e. that the housing conditions of the people affected need to be improved, many questions remain about how to do this. It is important to consider whether, once a dwelling has been renovated and basic sanitation needs have been met, without other changes, residents will be able to pay the increased overhead costs. It may also not be clear whose housing conditions should be improved, as in most cases low-quality housing is geographically clustered. Improving all the affected homes can be extremely costly, while selective refurbishment can lead to tensions within the community, creating external costs that may not have been anticipated. Furthermore, access to higher quality housing is not only available through renovation but also through moving. In this case, the potential impact of changes in the environment and the social network must also be taken into account. These are social policy dilemmas that are not easy to answer and solve. Programs aimed to improve housing conditions for the most deprived require careful planning and considerable expertise. However, it is also worth considering that investments in early childhood health are likely to pay off many times over in later life and can therefore significantly reduce the future costs of social security.

Our findings, based on high-quality administrative data, provide important evidence on the relationship between housing quality and the health of infants and young children, but they have limitations. Most importantly, we could estimate correlations, not causal relationships. Although we controlled for a number of factors, ranging from several characteristics of family background to time-invariant unobserved characteristics of the geographic microenvironment, there may still remain confounders that could be behind the observed relationship. Such factors may include health-related behaviors such as smoking, alcohol consumption or diet during pregnancy, the frequency of use of antenatal care, or young children's dietary habits. Of these unobserved confounders, maternal smoking during pregnancy and exposure to secondhand smoke are probably the most important. Unfortunately, our data do not contain information on these factors, but survey evidence suggests that even when smoking were included in the estimations, a sizeable association between poor housing quality and children's health would still remain (see Online Appendix B). In other words, the baseline control variables are likely to capture a large part of the variation in behavioral factors. Nevertheless, if we could control for all unobserved confounders, the estimated coefficients would be lower in absolute terms. At the same time, adverse conditions are likely to increase the likelihood of fetal losses, and these fetal losses are likely to disproportionately affect those in poor health, so fetuses who survive to live birth are positively selected^91,92^. This means that the association between poor housing quality and health may be somewhat underestimated in our analysis due to this selection issue. A finer measurement of housing quality would also be very useful. In developed countries, damp, moldy, or drafty dwellings may be an even more common problem than the indicators examined here. Temperature and humidity in the home may also be relevant for health. Finally, we would like to point out that although our study examined the relationship between housing quality and children's health, the effects of housing quality may be much broader than this. The lack of basic hygiene facilities and polluting heating can also affect outcomes, partly through health and partly independently, such as learning or general well-being, which may also have consequences for later adult life.

### Supplementary Information

Supplementary Information.

## Data Availability

The de-identified microdata sets used for this paper (live births, census and inpatient care records) are available for research purposes in the secure data environment of the HCSO (HCSO-CERS Research Room) following an accreditation process (in Budapest, Hungary). To access the data, contact: adatbank@krtk.hun-ren.hu.

## References

[1] Behrman JR, Rosenzweig MR (2004). Returns to birthweight. Rev. Econ. Stat..

[2] Black SE, Devereux PJ, Salvanes KG (2007). From the cradle to the labor market? The effect of birth weight on adult outcomes. Q. J. Econ..

[3] Case A, Fertig A, Paxson C (2005). The lasting impact of childhood health and circumstance. J. Health Econ..

[4] Smith JP (2009). The impact of childhood health on adult labor market outcomes. Rev. Econ. Stat..

[5] Currie J (2009). Healthy, wealthy, and wise: Socioeconomic status, poor health in childhood, and human capital development. J. Econ. Lit..

[6] Currie, J. & Almond, D. Human capital development before age five. In *Handbook of Labor Economics* (eds. Ashenfelter, O. & Card, D.) vol. 4, Part B 1315–1486 (Elsevier, 2011).

[7] Figlio D, Guryan J, Karbownik K, Roth J (2014). The effects of poor neonatal health on children’s cognitive development. Am. Econ. Rev..

[8] Bharadwaj P, Lundborg P, Rooth D-O (2018). Birth weight in the long run. J. Hum. Resour..

[9] Lambiris MJ (2022). Birth weight and adult earnings: A systematic review and meta-analysis. J. Dev. Orig. Health Dis..

[10] Karbownik K, Wray A (2022). Lifetime and intergenerational consequences of poor childhood health. J. Hum. Resour..

[11] Flores M, Wolfe BL (2022). Childhood health conditions and lifetime labor market outcomes. Am. J. Health Econ..

[12] Aizer A, Currie J (2014). The intergenerational transmission of inequality: Maternal disadvantage and health at birth. Science.

[13] Bilsteen JF, Andresen JB, Mortensen LH, Hansen AV, Andersen A-MN (2018). Educational disparities in perinatal health in Denmark in the first decade of the 21st century: A register-based cohort study. BMJ Open.

[14] Blumenshine P, Egerter S, Barclay CJ, Cubbin C, Braveman PA (2010). Socioeconomic disparities in adverse birth outcomes: A systematic review. Am. J. Prev. Med..

[15] Case A, Lubotsky D, Paxson C (2002). Economic status and health in childhood: The origins of the gradient. Am. Econ. Rev..

[16] Coffey D, Khera R, Spears D (2022). Mothers’ social status and children’s health: Evidence from joint households in rural India. Demography.

[17] Cook B, Wayne GF, Valentine A, Lessios A, Yeh E (2013). Revisiting the evidence on health and health care disparities among the Roma: A systematic review 2003–2012. Int. J. Public Health..

[18] Costa DL (2004). Race and pregnancy outcomes in the twentieth century: A long-term comparison. J. Econ. Hist..

[19] Hajdu T, Kertesi G, Kézdi G (2019). Health differences at birth between Roma and non-Roma children in Hungary: Long-run trends and decomposition. Popul. Dev. Rev..

[20] Martinson ML, Reichman NE (2016). Socioeconomic inequalities in low birth weight in the United States, the United Kingdom, Canada, and Australia. Am. J. Public Health..

[21] Mehta NK, Lee H, Ylitalo KR (2013). Child health in the United States: Recent trends in racial/ethnic disparities. Soc. Sci. Med..

[22] Oberg C, Colianni S, King-Schultz L (2016). Child health disparities in the 21st century. Curr. Probl. Pediatr. Adolesc. Health Care..

[23] Pillas D (2014). Social inequalities in early childhood health and development: A European-wide systematic review. Pediatr. Res..

[24] Szabó L, Boros J (2023). Socio-economic differences among low-birthweight infants in Hungary. Results of the Cohort ‘18—Growing Up in Hungary birth cohort study. PLoS One.

[25] Heckman JJ (2006). Skill formation and the economics of investing in disadvantaged children. Science.

[26] Karoly, L. A., Kilburn, M. R. & Cannon, J. S. *Early Childhood Interventions: Proven Results, Future Promise* (Rand Corporation, 2006).

[27] Chetty R, Hendren N, Katz LF (2016). The effects of exposure to better neighborhoods on children: New evidence from the moving to opportunity experiment. Am. Econ. Rev..

[28] Dherani M (2008). Indoor air pollution from unprocessed solid fuel use and pneumonia risk in children aged under five years: A systematic review and meta-analysis. Bull. World Health Organ..

[29] Fullerton DG, Bruce N, Gordon SB (2008). Indoor air pollution from biomass fuel smoke is a major health concern in the developing world. Trans. R. Soc. Trop. Med. Hyg..

[30] Fink G, Günther I, Hill K (2011). The effect of water and sanitation on child health: Evidence from the demographic and health surveys 1986–2007. Int. J. Epidemiol..

[31] Duflo, E., Greenstone, M., Guiteras, R. & Clasen, T. Toilets can work: Short and medium run health impacts of addressing complementarities and externalities in water and sanitation. 10.3386/w21521 (2015).

[32] Padhi BK (2015). Risk of adverse pregnancy outcomes among women practicing poor sanitation in rural India: A population-based prospective cohort study. PLoS Med..

[33] Freeman MC (2017). The impact of sanitation on infectious disease and nutritional status: A systematic review and meta-analysis. Int. J. Hyg. Environ. Health.

[34] Geruso M, Spears D (2018). Neighborhood sanitation and infant mortality. Am. Econ. J. Appl. Econ..

[35] Harville EW, Rabito FA (2018). Housing conditions and birth outcomes: The National Child Development Study. Environ. Res..

[36] Alsan M, Goldin C (2019). Watersheds in Child mortality: The role of effective water and sewerage infrastructure, 1880–1920. J. Polit. Econ..

[37] Headey D, Palloni G (2019). Water, sanitation, and child health: Evidence from subnational panel data in 59 countries. Demography.

[38] Lee KK (2020). Adverse health effects associated with household air pollution: A systematic review, meta-analysis, and burden estimation study. Lancet Glob. Health..

[39] Murray CJL (2020). Global burden of 87 risk factors in 204 countries and territories, 1990–2019: A systematic analysis for the Global Burden of Disease Study 2019. Lancet.

[40] Kinney PL (2021). Prenatal and postnatal household air pollution exposures and pneumonia risk: Evidence from the Ghana randomized air pollution and health study. Chest.

[41] Wolf J (2022). Effectiveness of interventions to improve drinking water, sanitation, and handwashing with soap on risk of diarrhoeal disease in children in low-income and middle-income settings: A systematic review and meta-analysis. Lancet.

[42] Younger A, Alkon A, Harknett K, Jean Louis R, Thompson LM (2022). Adverse birth outcomes associated with household air pollution from unclean cooking fuels in low- and middle-income countries: A systematic review. Environ. Res..

[43] Zhu L, Liao H, Burke PJ (2023). Household fuel transitions have substantially contributed to child mortality reductions in China. World Dev..

[44] Frostad JJ (2022). Mapping development and health effects of cooking with solid fuels in low-income and middle-income countries, 2000–18: A geospatial modelling study. Lancet Glob. Health.

[45] Prüss-Ustün A (2019). Burden of disease from inadequate water, sanitation and hygiene for selected adverse health outcomes: An updated analysis with a focus on low- and middle-income countries. Int. J. Hyg. Environ. Health..

[46] Wolf J (2023). Burden of disease attributable to unsafe drinking water, sanitation, and hygiene in domestic settings: A global analysis for selected adverse health outcomes. Lancet.

[47] Stoner O (2021). Household cooking fuel estimates at global and country level for 1990 to 2030. Nat. Commun..

[48] WHO & UNICEF (2021). Progress on Household Drinking Water, Sanitation and Hygiene 2000–2020: Five Years into the SDGs.

[49] Brown J (2023). The effects of racism, social exclusion, and discrimination on achieving universal safe water and sanitation in high-income countries. Lancet Glob. Health.

[50] Deitz S, Meehan K (2019). Plumbing poverty: Mapping hot spots of racial and geographic inequality in U.S. household water insecurity. Ann. Am. Assoc. Geograph..

[51] Ferguson L (2020). Exposure to indoor air pollution across socio-economic groups in high-income countries: A scoping review of the literature and a modelling methodology. Environ. Int..

[52] Mueller JT, Gasteyer S (2021). The widespread and unjust drinking water and clean water crisis in the United States. Nat. Commun..

[53] Anthonj C, Setty KE, Ezbakhe F, Manga M, Hoeser C (2020). A systematic review of water, sanitation and hygiene among Roma communities in Europe: Situation analysis, cultural context, and obstacles to improvement. Int. J. Hyg. Environ. Health..

[54] Filčák R, Škobla D (2021). Sanitation infrastructure at the systemic edge: Segregated Roma settlements and multiple health risks in Slovakia. Int. J. Environ. Res. Public Health..

[55] Kósa K, Daragó L, Ádány R (2011). Environmental survey of segregated habitats of Roma in Hungary: A way to be empowering and reliable in minority research. Eur. J. Public Health..

[56] Bíró A, Hajdu T, Kertesi G, Prinz D (2021). Life expectancy inequalities in Hungary over 25 years: The role of avoidable deaths. Popul. Stud..

[57] Mackenbach JP (2018). Trends in health inequalities in 27 European countries. Proc. Natl. Acad. Sci..

[58] Hegedüs, J. Hungary: Ideas and plans without political will. In *Social Housing in Transition Countries* (eds. Hegedüs, J., Lux, M. & Teller, N.) 180–194 (Routledge, 2013).

[59] Hegedüs, J. Social housing in Hungary. In *Social Housing in Europe* 205–221 (Wiley, 2014). 10.1002/9781118412367.ch12.

[60] Causa, O. & Pichelmann, J. *Should I Stay or Should I Go? Housing and Residential Mobility across OECD Countries*. (2020) 10.1787/d91329c2-en.

[61] Sánchez, A. C. & Andrews, D. Residential mobility and public policy in OECD countries. *OECD J. Econ. Stud. 2011*, (2011).

[62] Behrman RE (1985). Preventing low birth weight: A pediatric perspective. J. Pediatr..

[63] Birth P (2007). Causes, Consequences, and Prevention.

[64] Allinson JP (2023). Early childhood lower respiratory tract infection and premature adult death from respiratory disease in Great Britain: A national birth cohort study. Lancet.

[65] Pedersen M (2014). Ambient air pollution and pregnancy-induced hypertensive disorders. Hypertension.

[66] van den Hooven EH (2012). Air pollution exposure and markers of placental growth and function: The generation R study. Environ. Health Perspect..

[67] Ghosh R (2021). Ambient and household PM2.5 pollution and adverse perinatal outcomes: A meta-regression and analysis of attributable global burden for 204 countries and territories. PLoS Med..

[68] Molnár Á, Ádány R, Ádám B, Gulis G, Kósa K (2010). Health impact assessment and evaluation of a Roma housing project in Hungary. Health Place.

[69] Peat JK, Dickerson J, Li J (1998). Effects of damp and mould in the home on respiratory health: A review of the literature. Allergy.

[70] Mendell MJ, Mirer AG, Cheung K, Tong M, Douwes J (2011). Respiratory and allergic health effects of dampness, mold, and dampness-related agents: A review of the epidemiologic evidence. Environ. Health Perspect..

[71] Ingham T (2019). Damp mouldy housing and early childhood hospital admissions for acute respiratory infection: A case control study. Thorax.

[72] Pénzes J, Pásztor IZ, Tátrai P, Kóti T (2019). Roma population in Hungary—Spatial distribution and its temporal changes. DETUROPE Cent. Eur. J. Reg. Dev. Tour..

[73] Janky, B. The income situation of Gypsy families. In *Social Report 2004* (eds. Kolosi, T., Vukovich, G. & Tóth, I. G.) (TÁRKI, 2004).

[74] Kertesi G, Kézdi G (2011). The Roma/Non-Roma test score gap in Hungary. Am. Econ. Rev..

[75] Kertesi G, Kézdi G (2011). Roma employment in Hungary after the post-communist transition. Econ. Transit..

[76] Váradi, L. *Youths Trapped in Prejudice: Hungarian Adolescents’ Attitudes towards the Roma* (Springer Science & Business, 2014).

[77] Hajdu, T., Kertesi, G. & Kézdi, G. Inter-ethnic friendship and hostility between Roma and non-Roma students in Hungary: The role of exposure and academic achievement. *B. E. J. Econ. Anal. Policy.* **19**, (2019).

[78] Hajdu, T., Kertesi, G. & Kézdi, G. Ethnic segregation and inter-ethnic relationships in Hungarian schools. *Educ. J. Res. Debate* **4**, (2021).

[79] Scharle, Á. Schooling and employment of Roma youth: Changes between 2011 and 2016. In *The Hungarian Labor market 2020* (eds. Fazekas, K., Elek, P. & Hajdu, T.) 121–125 (Centre for Economic and Regional Studies-Institute of Economics, Budapest, 2021).

[80] Pope DP (2010). Risk of low birth weight and stillbirth associated with indoor air pollution from solid fuel use in developing countries. Epidemiol. Rev..

[81] Morisaki N, Kawachi I, Oken E, Fujiwara T (2017). Social and anthropometric factors explaining racial/ethnical differences in birth weight in the United States. Sci. Rep..

[82] Lhila A, Long S (2012). What is driving the black–white difference in low birthweight in the US?. Health Econ..

[83] Juárez SP, Hjern A (2017). The weight of inequalities: Duration of residence and offspring’s birthweight among migrant mothers in Sweden. Soc. Sci. Med..

[84] Aradhya S, Katikireddi SV, Juárez SP (2022). Immigrant ancestry and birthweight across two generations born in Sweden: An intergenerational cohort study. BMJ Glob. Health.

[85] Harding S, Santana P, Cruickshank JK, Boroujerdi M (2006). Birth weights of black african babies of migrant and nonmigrant mothers compared with those of babies of European mothers in Portugal. Ann. Epidemiol..

[86] Štípková M (2016). Immigrant disadvantage or the healthy immigrant effect? Evidence about low birth weight differences in the Czech Republic. Eur. J. Public Health..

[87] Singh GK, Yu SM (1996). Adverse pregnancy outcomes: Differences between US- and foreign-born women in major US racial and ethnic groups. Am. J. Public Health..

[88] Diez Roux AV, Mair C (2010). Neighborhoods and health. Ann. N. Y. Acad. Sci..

[89] Ellen IG, Mijanovich T, Dillman K-N (2001). Neighborhood effects on health: Exploring the links and assessing the evidence. J. Urban Aff..

[90] Shaw M (2004). Housing and public health. Annu. Rev. Public Health..

[91] Bruckner TA, Catalano R (2018). Selection in utero and population health: Theory and typology of research. SSM Popul. Health..

[92] Almond D, Currie J (2011). Killing me softly: The fetal origins hypothesis. J. Econ. Perspect..

