# Supplementary material for: Poor housing quality and the health of newborns and young children
Source: Sci Rep. 2024 Jun 5;14:12890. doi: 10.1038/s41598-024-63789-z (PMC11153610; doi:10.1038/s41598-024-63789-z)
Supplement: Supplementary file 1 — Supplementary Information. [file 41598_2024_63789_MOESM1_ESM.pdf]

Online Appendix

Appendix A: Figures and tables

Figure A1: Distribution of observations by values of the index of poor housing quality in the early childhood health sample

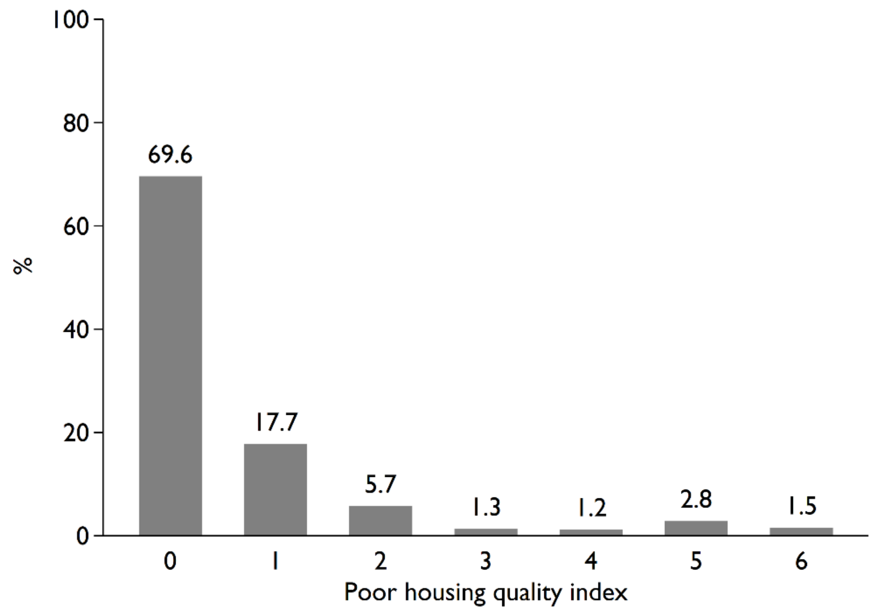

Notes: N=107,934.

Figure A2: Prevalence of the components of the poor housing quality index

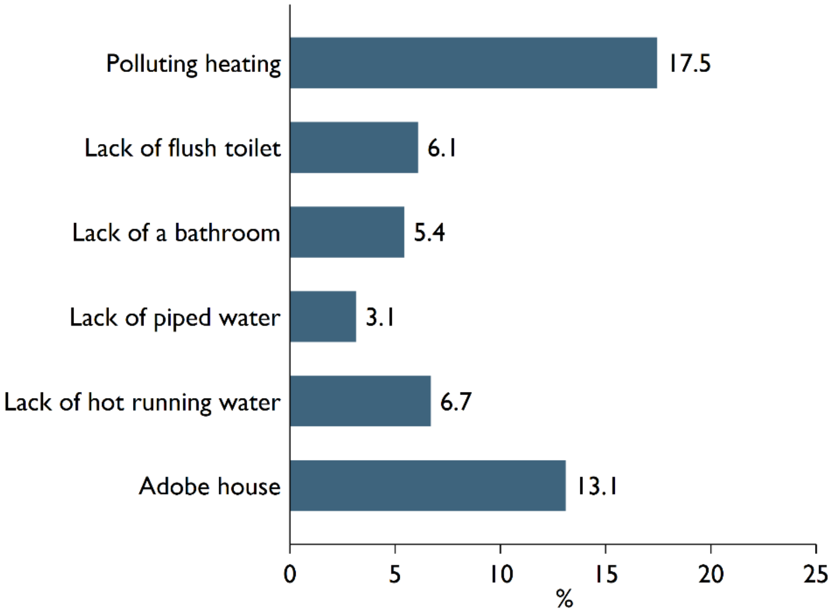

Notes: In the health at birth sample. N= 253,929.

Figure A3: Housing quality and health at birth, estimates using the six index items

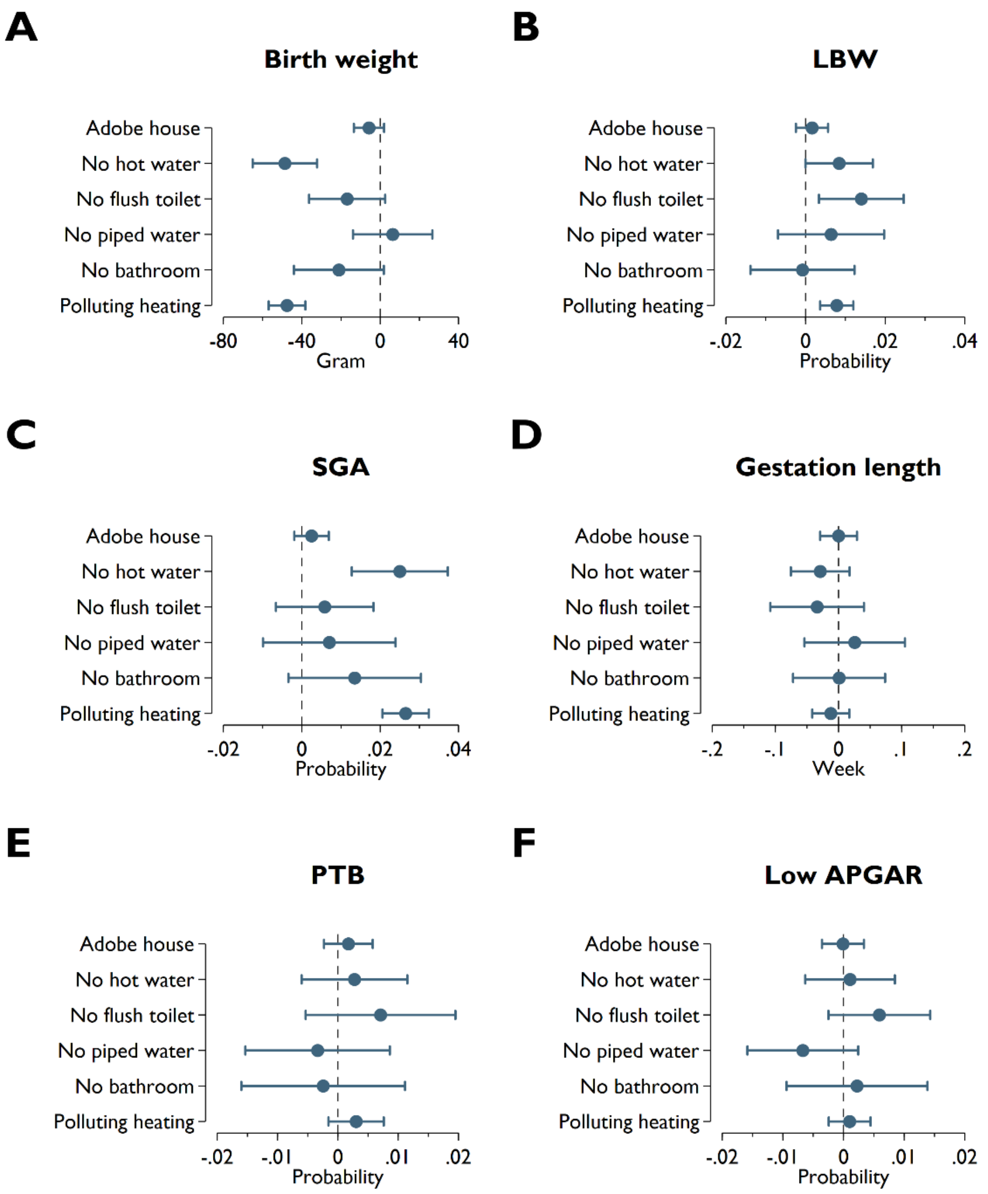

Notes: Each panel shows the result of a regression in which the six index items are used instead of the housing quality index. The dots show the estimated coefficients, the whiskers the 95% confidence intervals.

Figure A4: Housing quality and early childhood health, estimates using the six index items

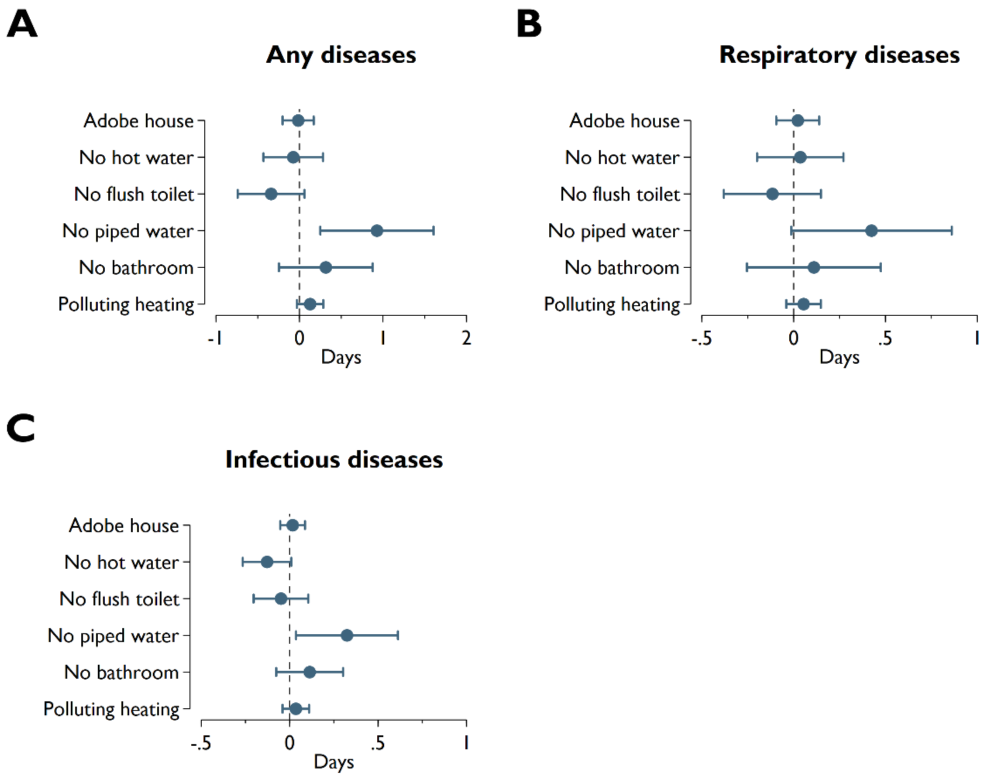

Notes: Each panel shows the result of a regression in which the six index items are used instead of the housing quality index. The dots show the estimated coefficients, the whiskers the 95% confidence intervals.

Table A1: N of observations by steps of sample selection, health at birth analysis sample

	N of observations	%
0. Total number of singleton births (2006/09-2011/08)	456,624	100.00%
1. Health-at-birth missing	−4,676	−1.02%
2. Unsuccessful link to Census	−44,480	−9.74%
3. Moved in after conception	−140,032	−30.67%
4. Housing quality indicators missing	−13,507	−2.96%
Final sample	253,929	55.61%

Table A2: N of observations by steps of sample selection, early childhood health analysis sample

	N of observations	%
0. Total number of singleton births (2008/01-2011/08)	330,627	100.00%
1. Health-at-birth missing	−2,935	−0.89%
2. Unsuccessful link to Census	−32,227	−9.75%
3. Moved in after conception	−88,447	−26.75%
4. Housing quality indicators missing	−10,290	−3.11%
5. Early childhood health missing	−88,794	−26.86%
Final sample	107,934	32.65%

Table A3: Mean health outcomes at birth by steps of sample selection

Outcome	Singleton births full sample (N = 456,385)	Health at birth analysis sample (N = 253,929)	Early childhood health analysis sample (N = 107,934)
Birth weight	3306 (546)	3327 (541)	3317 (545)
LBW	0.063 (0.243)	0.057 (0.233)	0.061 (0.238)
SGA	0.106 (0.307)	0.098 (0.297)	0.102 (0.303)
Gestation length	38.8 (1.7)	38.8 (1.7)	38.8 (1.7)
PTB	0.068 (0.252)	0.065 (0.247)	0.068 (0.251)
low APGAR	0.054 (0.226)	0.052 (0.222)	0.056 (0.230)

Notes: the table reports the evolution of the key health outcomes' mean and standard deviation at different steps of sample selection. Standard deviations are in parentheses.

Table A4: Descriptive statistics of the control variables

	Health at birth sample (N=253,929)		Early childhood health sample (N=107,934)	
**Variable**	**Mean**	**SD**	**Mean**	**SD**
Female	0.487	0.500	0.487	0.500
Mother’s age				
13-17	0.017	0.130	0.019	0.136
18-24	0.146	0.354	0.165	0.371
25-29	0.294	0.456	0.294	0.456
30-34	0.370	0.483	0.355	0.479
35-39	0.147	0.354	0.142	0.349
40+	0.026	0.158	0.025	0.157
Mother’s education				
primary or less	0.175	0.380	0.196	0.397
vocational	0.159	0.366	0.175	0.380
high school	0.336	0.472	0.335	0.472
tertiary	0.324	0.468	0.289	0.453
missing	0.006	0.080	0.006	0.077
Mother’s marital status				
single	0.310	0.462	0.326	0.469
married	0.641	0.480	0.625	0.484
widow	0.003	0.057	0.003	0.057
divorced	0.046	0.208	0.046	0.210
Mother’s labor market status				
active	0.668	0.471	0.641	0.480
on maternity leave	0.174	0.379	0.185	0.388
unemployed	0.056	0.231	0.069	0.253
student	0.017	0.131	0.019	0.136
other	0.076	0.264	0.079	0.270
missing	0.008	0.092	0.007	0.086
Mother’s ethnicity				
Roma	0.069	0.254	0.078	0.269
N of previous live births				
0	0.425	0.494	0.414	0.493
1	0.359	0.480	0.360	0.480
2	0.137	0.344	0.142	0.349
3	0.045	0.207	0.048	0.214
4	0.019	0.135	0.020	0.139
5+	0.015	0.122	0.016	0.127
N of previous induced abortions				

0	0.838	0.368	0.836	0.370
1	0.117	0.321	0.119	0.324
2	0.032	0.175	0.031	0.173
3+	0.013	0.115	0.014	0.117
N of previous spontaneous pregnancy losses				
0	0.848	0.359	0.844	0.362
1	0.119	0.323	0.122	0.327
2	0.025	0.157	0.026	0.158
3+	0.008	0.089	0.008	0.092
Father's age				
13-17	0.001	0.036	0.001	0.038
18-24	0.053	0.224	0.060	0.238
25-29	0.174	0.379	0.173	0.378
30-34	0.361	0.480	0.354	0.478
35-39	0.216	0.412	0.215	0.411
40+	0.109	0.312	0.107	0.310
missing	0.086	0.280	0.089	0.285
Father's education				
primary or less	0.113	0.317	0.129	0.335
vocational	0.268	0.443	0.294	0.456
high school	0.281	0.450	0.275	0.447
tertiary	0.241	0.428	0.202	0.402
missing	0.097	0.295	0.100	0.300
Father's labor market status				
active	0.813	0.390	0.796	0.403
on maternity leave	0.000	0.020	0.000	0.020
unemployed	0.055	0.228	0.064	0.245
student	0.003	0.056	0.003	0.057
other	0.029	0.167	0.034	0.180
missing	0.100	0.300	0.103	0.303
Father's ethnicity				
Roma	0.056	0.230	0.063	0.244
missing	0.104	0.305	0.107	0.309

Notes: Occupations codes, census tracts, and birth-year-by-birth-month information are not shown due to their large number.

Table A5: Housing quality and health at birth, ZIP code fixed effects

	(1) Birth weight	(2) LBW	(3) SGA	(4) Gestation length	(5) PTB	(6) Low APGAR
Poor housing quality index	−24.76***	0.0066***	0.0141***	−0.0103**	0.0018***	0.0011*
	(1.23)	(0.0007)	(0.0010)	(0.0041)	(0.0006)	(0.0007)
N of obs.	253,929	253,929	253,929	253,929	253,929	253,929
R-squared	0.111	0.053	0.065	0.053	0.040	0.260
Controls	Yes	Yes	Yes	Yes	Yes	Yes
ZIP code FE	Yes	Yes	Yes	Yes	Yes	Yes
Year-by-month FE	Yes	Yes	Yes	Yes	Yes	Yes

Notes: Controls: sex of the child, the highest level of education, labor market status, occupation code, ethnicity, and age of the mother and father, marital status of the mother, number of previous live births, induced abortions, and spontaneous fetal losses of the mother. Robust standard errors are in parentheses. *** p<0.01, ** p<0.05, * p<0.1.

Table A6: Housing quality and health at birth, additional controls

	(1) Birth weight	(2) LBW	(3) SGA	(4) Gestation length	(5) PTB	(6) Low APGAR
Poor housing quality index	−22.44***	0.0063***	0.0129***	−0.0122**	0.0021***	0.0089
	(1.40)	(0.0007)	(0.0010)	(0.0047)	(0.0007)	(0.0006)
N of obs.	253,929	253,929	253,929	253,929	253,929	253,929
R-squared	0.129	0.071	0.082	0.072	0.057	0.275
Controls	Yes	Yes	Yes	Yes	Yes	Yes
Additional controls	Yes	Yes	Yes	Yes	Yes	Yes
Census tract FE	Yes	Yes	Yes	Yes	Yes	Yes
Year-by-month FE	Yes	Yes	Yes	Yes	Yes	Yes

Notes: Controls: sex of the child, the highest level of education, labor market status, occupation code, ethnicity, and age of the mother and father, marital status of the mother, number of previous live births, induced abortions, and spontaneous fetal losses of the mother. Additional controls: the number of household members of different ages, the proportion of employed persons among 25-59-year-olds, the proportion of tertiary and secondary education among 25-59-year-olds, the proportion of people speaking foreign languages (English, German) among 25-59-year-olds, the proportion of people with long-lasting disease or impairment among 25-59-year-olds, and floor space per inhabitant in the dwelling. Robust standard errors are in parentheses. *** p<0.01, ** p<0.05, * p<0.1.

Table A7: Housing quality and health at birth, weighted regressions

	(1)<br>Birth weight	(2)<br>LBW	(3)<br>SGA	(4)<br>Gestation length	(5)<br>PTB	(6)<br>Low APGAR
Poor housing quality index	−24.64***<br>(1.41)	0.0062***<br>(0.0008)	0.0141***<br>(0.0011)	−0.0125**<br>(0.0048)	0.0018**<br>(0.0007)	0.0010<br>(0.0007)
N of obs.	253,929	253,929	253,929	253,929	253,929	253,929
R-squared	0.135	0.075	0.086	0.077	0.061	0.282
Controls	Yes	Yes	Yes	Yes	Yes	Yes
Census tract FE	Yes	Yes	Yes	Yes	Yes	Yes
Year-by-month FE	Yes	Yes	Yes	Yes	Yes	Yes

Notes: Inverse probability weights are applied to account for selection. Controls: sex of the child, the highest level of education, labor market status, occupation code, ethnicity, and age of the mother and father, marital status of the mother, number of previous live births, induced abortions, and spontaneous fetal losses of the mother. Robust standard errors are in parentheses. *** p<0.01, ** p<0.05, * p<0.1.

Table A8: Housing quality and health at birth, narrower sample

	(1)<br>Birth weight	(2)<br>LBW	(3)<br>SGA	(4)<br>Gestation length	(5)<br>PTB	(6)<br>Low APGAR
Poor housing quality index	−23.00***<br>(1.71)	0.0059***<br>(0.0010)	0.0137***<br>(0.0013)	−0.0042<br>(0.0056)	0.0015*<br>(0.0007)	0.0009<br>(0.0006)
N of obs.	164,943	164,943	164,943	164,943	164,943	164,943
R-squared	0.145	0.086	0.102	0.087	0.073	0.276
Controls	Yes	Yes	Yes	Yes	Yes	Yes
Census tract FE	Yes	Yes	Yes	Yes	Yes	Yes
Year-by-month FE	Yes	Yes	Yes	Yes	Yes	Yes

Notes: Births between September 2008 and August 2011. Controls: sex of the child, the highest level of education, labor market status, occupation code, ethnicity, and age of the mother and father, marital status of the mother, number of previous live births, induced abortions, and spontaneous fetal losses of the mother. Robust standard errors are in parentheses. *** p<0.01, ** p<0.05, * p<0.1.

Table A9: Housing quality and health at birth, using sum of the z-scores of the components

	(1)<br>Birth weight	(2)<br>LBW	(3)<br>SGA	(4)<br>Gestation length	(5)<br>PTB	(6)<br>Low APGAR
Poor housing quality index (based on z-scores)	−5.96***<br>(0.36)	0.0016***<br>(0.0002)	0.0035***<br>(0.0003)	−0.0026**<br>(0.0012)	0.00042**<br>(0.00017)	0.00026*<br>(0.00015)
N of obs.	253,929	253,929	253,929	253,929	253,929	253,929
R-squared	0.128	0.070	0.071	0.057	0.275	0.082
Controls	Yes	Yes	Yes	Yes	Yes	Yes
Census tract FE	Yes	Yes	Yes	Yes	Yes	Yes
Year-by-month FE	Yes	Yes	Yes	Yes	Yes	Yes

Notes: Controls: sex of the child, the highest level of education, labor market status, occupation code, ethnicity, and age of the mother and father, marital status of the mother, number of previous live births, induced abortions, and spontaneous fetal losses of the mother. Robust standard errors are in parentheses. *** p<0.01, ** p<0.05, * p<0.1.

Table A10: Housing quality and health at birth, log-transformed outcome variables

	(1) ln(Birth weight)	(2) ln(Gestation length)
Poor housing quality index	−0.0078***<br>(0.0005)	−0.0003**<br>(0.0001)
N of obs.	253,929	253,929
R-squared	0.118	0.070
Controls	Yes	Yes
Census tract FE	Yes	Yes
Year-by-month FE	Yes	Yes

Notes: Controls: sex of the child, the highest level of education, labor market status, occupation code, ethnicity, and age of the mother and father, marital status of the mother, number of previous live births, induced abortions, and spontaneous fetal losses of the mother. Robust standard errors are in parentheses. *** p<0.01, ** p<0.05, * p<0.1.

Table A11: Housing quality and early childhood health, ZIP code fixed effects

	(1) Any diseases	(2) Respiratory diseases	(3) Infectious diseases
Poor housing quality index	0.113***<br>(0.038)	0.067**<br>(0.025)	0.036**<br>(0.016)
N of obs.	107,934	107,934	107,934
R-squared	0.053	0.069	0.049
Controls	Yes	Yes	Yes
ZIP code FE	Yes	Yes	Yes
Year-by-month FE	Yes	Yes	Yes

Notes: The dependent variables are the number of days spent in inpatient care at the age of 1-2 years. Respiratory diseases = ICD-10 codes J00-J99. Infectious diseases = ICD-10 codes A00-B99. Controls: sex of the child, the highest level of education, labor market status, occupation code, ethnicity, and age of the mother and father, marital status of the mother, number of previous live births, induced abortions, and spontaneous fetal losses of the mother. Robust standard errors are in parentheses. *** p<0.01, ** p<0.05, * p<0.1.

Table A12: Housing quality and early childhood health, additional controls

	(1)<br>Any diseases	(2)<br>Respiratory diseases	(3)<br>Infectious diseases
Poor housing quality index	0.099***<br>(0.036)	0.063**<br>(0.027)	0.031**<br>(0.015)
N of obs.	107,934	107,934	107,934
R-squared	0.081	0.092	0.077
Controls	Yes	Yes	Yes
Additional controls	Yes	Yes	Yes
Census tract FE	Yes	Yes	Yes
Year-by-month FE	Yes	Yes	Yes

Notes: The dependent variables are the number of days spent in inpatient care at the age of 1-2 years. Respiratory diseases = ICD-10 codes J00-J99. Infectious diseases = ICD-10 codes A00-B99. Controls: sex of the child, the highest level of education, labor market status, occupation code, ethnicity, and age of the mother and father, marital status of the mother, number of previous live births, induced abortions, and spontaneous fetal losses of the mother. Additional controls: the number of household members of different ages, the proportion of employed persons among 25-59-year-olds, the proportion of tertiary and secondary education among 25-59-year-olds, the proportion of people speaking foreign languages (English, German) among 25-59-year-olds, the proportion of people with long-lasting disease or impairment among 25-59-year-olds, and floor space per inhabitant in the dwelling. Robust standard errors are in parentheses. *** p<0.01, ** p<0.05, * p<0.1.

Table A13: Housing quality and early childhood health, weighted regressions

	(1)<br>Any diseases	(2)<br>Respiratory diseases	(3)<br>Infectious diseases
Poor housing quality index	0.091**<br>(0.038)	0.061**<br>(0.030)	0.031*<br>(0.016)
N of obs.	107,934	107,934	107,934
R-squared	0.090	0.099	0.082
Controls	Yes	Yes	Yes
Census tract FE	Yes	Yes	Yes
Year-by-month FE	Yes	Yes	Yes

Notes: Inverse probability weights are applied to account for selection. The dependent variables are the number of days spent in inpatient care at the age of 1-2 years. Respiratory diseases = ICD-10 codes J00-J99. Infectious diseases = ICD-10 codes A00-B99. Controls: sex of the child, the highest level of education, labor market status, occupation code, ethnicity, and age of the mother and father, marital status of the mother, number of previous live births, induced abortions, and spontaneous fetal losses of the mother. Robust standard errors are in parentheses. *** p<0.01, ** p<0.05, * p<0.1.

Table A14: Housing quality and early childhood health, using sum of the z-scores of the components

	(1)<br>Any diseases	(2)<br>Respiratory diseases	(3)<br>Infectious diseases
Poor housing quality index (based on z-scores)	0.030***<br>(0.009)	0.018**<br>(0.007)	0.009**<br>(0.004)
N of obs.	107,934	107,934	107,934
R-squared	0.081	0.092	0.077
Controls	Yes	Yes	Yes
Census tract FE	Yes	Yes	Yes
Year-by-month FE	Yes	Yes	Yes

Notes: The dependent variables are the number of days spent in inpatient care at the age of 1-2 years. Respiratory diseases = ICD-10 codes J00-J99. Infectious diseases = ICD-10 codes A00-B99. Controls: sex of the child, the highest level of education, labor market status, occupation code, ethnicity, and age of the mother and father, marital status of the mother, number of previous live births, induced abortions, and spontaneous fetal losses of the mother. Robust standard errors are in parentheses. *** p<0.01, ** p<0.05, * p<0.1.

Table A15: Housing quality and early childhood health, controls for health at birth

	(1)<br>Any diseases	(2)<br>Respiratory diseases	(3)<br>Infectious diseases
Poor housing quality index	0.095**<br>(0.036)	0.062**<br>(0.026)	0.031**<br>(0.015)
N of obs.	107,934	107,934	107,934
R-squared	0.085	0.095	0.078
Controls	Yes	Yes	Yes
Health at birth	Yes	Yes	Yes
Census tract FE	Yes	Yes	Yes
Year-by-month FE	Yes	Yes	Yes

Notes: The dependent variables are the number of days spent in inpatient care at the age of 1-2 years. Respiratory diseases = ICD-10 codes J00-J99. Infectious diseases = ICD-10 codes A00-B99. Controls: sex of the child, the highest level of education, labor market status, occupation code, ethnicity, and age of the mother and father, marital status of the mother, number of previous live births, induced abortions, and spontaneous fetal losses of the mother. Control variables for health at birth: birth weight, gestation length, low APGAR score, SGA. Robust standard errors are in parentheses. *** p<0.01, ** p<0.05, * p<0.1.

Table A16: Estimated change in health at birth when PHQI is improved (in SD units)

	Birth weight	LBW	SGA	Gestation length	PTB	Low APGAR
Mean of outcome	3327.3	0.0575	0.0978	38.85	0.0653	0.0522
SD of outcome	541.4	0.2328	0.2970	1.71	0.2470	0.2223
Coefficient	−24.39	0.0064	0.0140	−0.0106	0.0018	0.0011
Change when PHQI is improved by						
6	0.27	−0.16	−0.28	0.04	−0.04	−0.03
5	0.23	−0.14	−0.24	0.03	−0.04	−0.02
4	0.18	−0.11	−0.19	0.02	−0.03	−0.02
3	0.14	−0.08	−0.14	0.02	−0.02	−0.01
2	0.09	−0.05	−0.09	0.01	−0.01	−0.01
1	0.05	−0.03	−0.05	0.01	−0.01	0.00

Notes: Change is measured in units of SD of outcome. The coefficients are from Table 2.

Table A17: Estimated change in early childhood health when PHQI is improved (in SD units)

	Any diseases	Respiratory diseases	Infectious diseases
Mean of outcome	1.90	0.90	0.59
SD of outcome	7.39	4.21	3.27
Coefficient	0.108	0.067	0.034
Change when PHQI is improved by			
6	−0.09	−0.10	−0.06
5	−0.07	−0.08	−0.05
4	−0.06	−0.06	−0.04
3	−0.04	−0.05	−0.03
2	−0.03	−0.03	−0.02
1	−0.01	−0.02	−0.01

Notes: Change is measured in units of SD of outcome. The coefficients are from Table 3.

Appendix B: On the potential confounding effects of smoking

This brief analysis examines how accounting for maternal smoking and exposure to secondhand smoke might change the coefficient of poor housing quality. For this exercise, we use a survey of young children. The National Children's Respiratory Survey, conducted by the National Institute of Environmental Health in 2017, covers more than 60,000 children in grade 3. This survey includes most of our key control variables, some but not all of our outcome variables and only one indicator of poor housing quality (polluting heating). In this sense, this database is limited in its ability to answer only our main research questions, but it has the great advantage of containing information on maternal smoking during pregnancy and fetal exposure to secondhand smoke.

We can examine four outcome variables: (i) severe lower respiratory tract disease in age <2 years, (ii) birth weight, (iii) low birth weight (<2500 grams), (iv) pre-term birth (pregnancy length ≤37 weeks). Note that the definition of pre-term birth is different from that in the main analysis, as pregnancy length is given as a categorical variable in the survey.

Poor housing quality is captured by polluting heating, measured in a similar way as in the main analysis (solid fuel heating and separate heating of each room).

Maternal smoking during pregnancy was not simply asked as a yes/no question, but the average number of cigarettes smoked per day is also known. Fetal exposure to secondhand smoke in the home was captured by a binary variable.

The questionnaire also included a question on whether the child had lived in the same home since birth. This allowed us to exclude those who had moved. In this way, the housing conditions measured in 2017 are more likely to resemble the housing conditions experienced while in the womb. The final sample consists of about 33,000 children.

We estimated two regressions that examine the relationship between polluting heating and the health at birth and at age <2 years. One with the baseline control variables (age, education, Roma ethnicity and labor market status of the mother and father, sex of the child), birth-year-by-birth-month fixed effects and ZIP code fixed effects. And another one that includes, in addition to these, information on maternal smoking during pregnancy and fetal exposure to secondhand smoke. We are interested in the change in the coefficient of poor housing between

the two regressions. Does the inclusion of smoking significantly change the coefficient of polluting heating?

Note that this empirical design is not identical to the empirical design of the main analysis. First, some control variables are not included in this survey (pregnancy history of the mother, marital status, detailed occupation categories). Therefore, the inclusion of smoking may induce a larger change in the coefficients of interest than would be observed in our main analysis. Second, the outcome variables are measured by retrospective questions, which may affect the precision of the estimates. In addition, only a non-standard measure of preterm birth can be produced from the survey. Third, we cannot reproduce our poor housing quality index, we only use one item. Fourth, housing conditions are measured several years later than when the children were in the womb. Over these years, they may have changed in some cases. Fifth, the sample is a fraction of the sample used in the main analysis, so the precision of the estimates is much lower and the standard errors much larger than would be seen in a larger sample. In sum, in these analyses, it is not the point estimates themselves and their statistical significance that are of interest, but the changes in the point estimates as a result of controlling for smoking.

Figure B1 summarizes the results. Circles show the point estimates without smoking, while squares show the point estimates with smoking control. Taking into account and bearing in mind the differences and shortcomings, these results suggest that the associations between poor housing quality and newborn and early childhood health do not disappear even when maternal smoking during pregnancy and exposure to secondhand smoke during fetal life are controlled.

Figure B1: Polluting heating, health at birth and early childhood health – the distorting effect of smoking

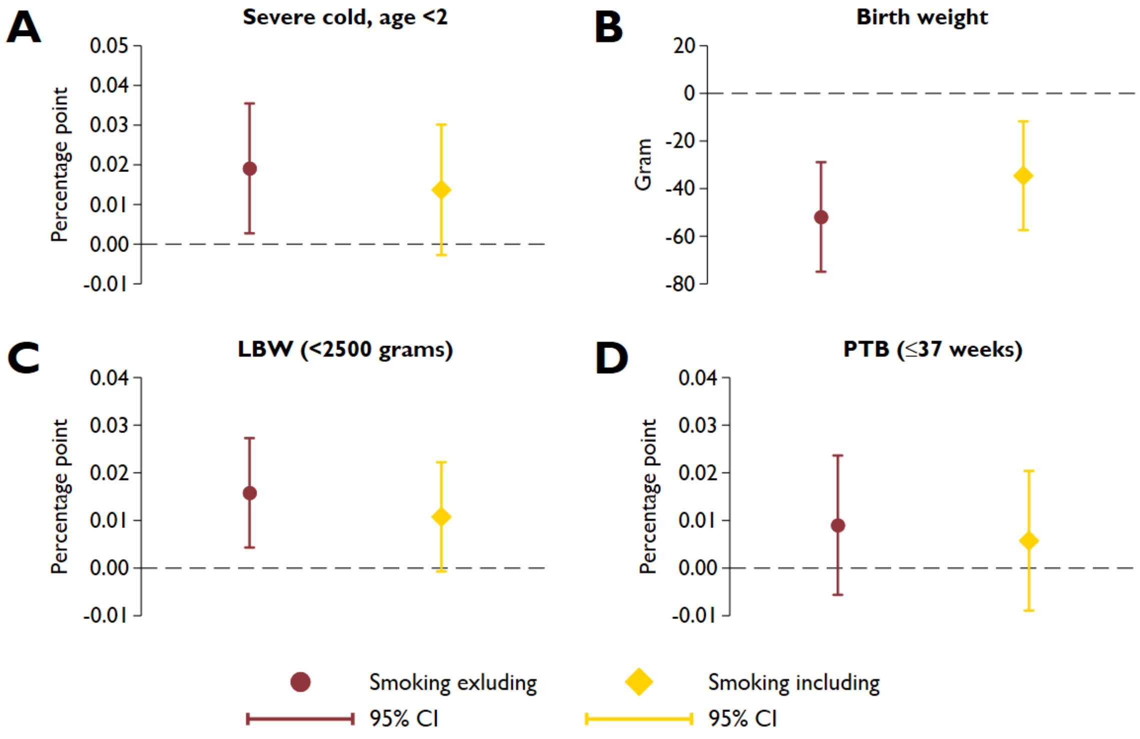

Notes: The circles and squares show the coefficients of the indicators of poor housing quality. Controls: sex of the child, the highest level of education, labor market status, ethnicity, and age of the mother and father. Year-by-month fixed effects and ZIP code fixed effects are included in the models. The error bars represent 95% confidence intervals.
